# Impact of e-publication changes in the International Code of Nomenclature for algae, fungi and plants (Melbourne Code, 2012) - did we need to “run for our lives”?

**DOI:** 10.1186/s12862-017-0961-8

**Published:** 2017-05-25

**Authors:** Nicky Nicolson, Katherine Challis, Allan Tucker, Sandra Knapp

**Affiliations:** 1 Biodiversity Informatics & Spatial Analysis, Royal Botanic Gardens, Kew, Richmond Surrey, TW9 3AA UK; 2 IPNI, Royal Botanic Gardens, Kew, Richmond Surrey, TW9 3AA UK; 3 Department of Computer Science, Brunel University London, Uxbridge, UB8 3PH UK; 4 Department of Life Sciences, Natural History Museum, Cromwell Road, London, SW7 5BD UK

**Keywords:** Publishing, On-line, Botany, Nomenclature, Taxonomy

## Abstract

**Background:**

At the Nomenclature Section of the XVIII International Botanical Congress in Melbourne, Australia (IBC), the botanical community voted to allow electronic publication of nomenclatural acts for algae, fungi and plants, and to abolish the rule requiring Latin descriptions or diagnoses for new taxa. Since the 1st January 2012, botanists have been able to publish new names in electronic journals and may use Latin or English as the language of description or diagnosis.

**Results:**

Using data on vascular plants from the International Plant Names Index (IPNI) spanning the time period in which these changes occurred, we analysed trajectories in publication trends and assessed the impact of these new rules for descriptions of new species and nomenclatural acts. The data show that the ability to publish electronically has not “opened the floodgates” to an avalanche of sloppy nomenclature, but concomitantly neither has there been a massive expansion in the number of names published, nor of new authors and titles participating in publication of botanical nomenclature.

**Conclusions:**

The e-publication changes introduced in the Melbourne Code have gained acceptance, and botanists are using these new techniques to describe and publish their work. They have not, however, accelerated the rate of plant species description or participation in biodiversity discovery as was hoped.

**Electronic supplementary material:**

The online version of this article (doi:10.1186/s12862-017-0961-8) contains supplementary material, which is available to authorized users.

## Background

Publication of results is one of the cornerstones of the scientific endeavour. Differences between scientific and general publishing were first articulated by Henry Oldenburg, who as Secretary of the Royal Society, established the first English-language scientific journal, Philosophical Transactions of the Royal Society [1]. Oldenberg’s functions for scientific publication were dissemination, registration, certification and archiving (called by him the “Minutes of Science”); scientific publishing therefore has a role in informing not only in the present, but also for future generations. Scientific (scholarly) publication has seen great change driven in part by increased interconnectivity of research communities, massive increases in funding for research and development since the middle of the 20th century, and key technological advances such as the Internet. These drivers are characterised as having as big an effect as the replacement of parchment by paper, or the advent of mass printing technologies [2]. Moves away from print on paper to electronic-only publishing parallel increasing scientific activity on-line, and the pace of change in this area of scientific publishing is increasing, with more and more journals converting to on-line only publishing (e.g., Evolution, New Phytologist, Biological Journal of the Linnean Society).

“Published work” has a central place in nomenclature (the scientific naming of organisms), and until January 2012, nomenclatural acts published in electronic-only form were not considered valid/effective (see [1, 3, 4]) leading many to consider the taxonomic community as distinctly behind the curve relative to the rest of the scientific community. Discussions about publication went on in both the zoological and botanical (those working on algae, fungi and plants) communities, but largely separately, since the two rulebooks for naming (Codes of nomenclature) are governed very differently (see [5] for a history of the Codes), although many of the central issues were the same for both. Here we treat only e-publication as it pertains to algae, fungi and plants, whose nomenclature rules are contained in the current *International Code of Nomenclature for algae, fungi and plants* [6], hereafter referred to as the ICN or Melbourne Code. Decisions about changes to the rules of naming for this community are made at Nomenclature Sections of International Botanical Congresses (IBC) held every six years [7, 8].

Discussions about electronic publication in the botanical community began in the 1990s, formal proposals at the 1999 XVI IBC in St Louis [9] and at the XVII IBC in Vienna [10] to allow e-publication were defeated, but suggestions about e-publication were included in the Vienna Code [11, 12]. Issues arising were largely those of archiving, accessibility and tracking dates of publication; this last is critical, because the principle of priority that is one of the pillars of nomenclature depends upon accurate knowledge of date of publication (see [8] for an explanation of the principle of priority). A Special Committee was established at the Vienna Congress to examine the issues, with the mandate to prepare proposals for the next IBC in Melbourne in 2011 [13]. Over the six years between the XVII (Vienna) and XVIII (Melbourne) Congresses, publication rules were challenged by Knapp [14], who published new species in PLoS ONE - an on-line only journal - and complied with letter of the Code by depositing ten offprints in botanical libraries [15].

30A.2. To aid availability through time and place, authors publishing nomenclatural novelties should give preference to periodicals that regularly publish taxonomic articles, or else printed copies of a publication (even if also distributed electronically) should be deposited in at least ten, but preferably more, botanical or other generally accessible libraries throughout the world including a name-indexing centre appropriate to the taxonomic group. [11].

This posed a significant cataloguing and preservation challenge for libraries, who felt they might be facing a deluge of single- or few-page paper copies of papers describing new species [15] (also see Doug Holland conference presentation: “Libraries and the Code: The changing role of botanical libraries in the age of electronic publication.”, Biodiversity Information Standards (TDWG) 2011). Proposals put forward by the Special Committee on Electronic Publication [16] to allow e-publication under the then “botanical code” were accepted overwhelmingly at the Nomenclature Section of the XVIII IBC in Melbourne Australia in July 2011 [17, 18]. At the same time, proposals to change the rules that required a description or diagnosis of new taxa to be in Latin were also accepted [18, 19]. Changes to the rules of naming usually come into force two years after the IBC, but such was the excitement of many in the community for change that these two major changes were voted to come into force in January 2012, a year earlier than “normal” [20]. About six months later, the zoological Commissioners voted to accept e-publication of new names and nomenclatural acts for animals [4], and backdated their new rule to January 2012 to harmonise dates. One major difference in the implementation of e-publication in the two communities is that in zoology, e-publication must be accompanied by registration in ZooBank (www.zoobank.org; for description of ZooBank see [21], while for algae, fungi and plants, e-publication is only another publication type and is not necessarily linked to registration. Fungal names, however, must be registered to be validly published [6].

The advent of e-publication for nomenclatural acts for algae, fungi and plants was both welcomed and feared (see Table 2). Tracking the realization of these effects is difficult for many groups of organisms, but with vascular plants, we have a unique opportunity to conduct an analysis using data from the International Plant Names Index (IPNI, www.ipni.org) which records new names and combinations (generic reassignments, see [8]) for these taxa. IPNI began as Index Kewensis, which was started with a £250 legacy from Charles Darwin in his will for the “establishment of an index of all plants” [22, 23]. It was conceived in a time when it was feasible for a scientist to own all the relevant literature for their field, but it was even then necessary to have a bibliographic index to avoid repeated reference to scattered primary sources. The Index captured the name, authorship and basic bibliographic details of published plant names. In 1983 the data were digitised to an electronic database format, and in the late 1990s Index Kewensis was amalgamated with the Gray Card Index (GCI) maintained by the Harvard University Herbaria and the Australian Plant Names Index (APNI) to form the International Plant Names Index (IPNI, www.ipni.org see [22]). This dataset is accessible online and is continuously updated by a dedicated editorial team as new names are published; approximately 8000 new name records are added each year. The dataset is a valuable resource for trends analysis regarding the time, location and method of publication of new plant names.

In this paper, we analyse publication trajectories for nomenclature governed by the ICN [6] using data from IPNI to examine whether the hopes-increased participation, increased rate of description-or fears-avalanche of sloppy nomenclature, proliferation of new on-line journals - have been realised. It is not our intention to review the debates on e-publication in taxonomy here, nor are we comparing the effects of the changes in the rules between zoology and botany (algae, fungi and plants). Problems with the new rules have been highlighted by some [24, 25], and within the community working with algae, fungi and plants, new changes to improve the rules surrounding e-publication continue to be proposed [26]. These will be discussed at the Nomenclature Section of the XIX IBC in Shenzhen, China in July 2017 (Shenzen XIX IBC).

## Methods

### Where the data are from and how they were recorded

The IPNI database contains basic bibliographic information about the place of first publication of vascular plant names (ferns and fern allies, conifers, cycads and flowering plants). Nomenclatural acts representing new names, new combinations, replacement names and names at new ranks are recorded, with the date of effective publication. Note that lectotypifications are also nomenclatural acts which may be published electronically, but these are not included in this analysis. See [6] and [8] for definitions of nomenclatural acts. This dataset does not include nomenclatural acts in algae or fungi, also governed by the same rules as vascular plants.

The authorship of the nomenclatural act is standardised using the principles laid out in Authors of Plant Names [27] (also referenced under recommendation 46A of the ICN [6]). Publication titles are also standardised by linking to an authoritative list. The set of data recorded for each nomenclatural act has been expanded since the changes in the Melbourne Code came into effect (1st January 2012), to include:

- publication channel: to indicate if the work was published on paper or as an e-publication. This is set to e-publication if the article containing the nomenclatural act is either published online before print or is published online only. The default value of the flag indicates paper publication.
- language: indicates that the description or diagnosis is written in English. The default value of the flag indicates use of Latin language.
- Digital Object Identifiers (DOIs) - these can be resolved to access metadata about the publication and to navigate to the reference online (if available).

Members of the editorial team apply the rules of the ICN and exercise nomenclatural judgement about the nomenclatural acts recorded. Annotations to indicate if an act is illegitimate, not effectively published, or not validly published are added to that nomenclatural act record.

The records are fully versioned, with date of application of each edit recorded.

### Selection of data subset for analysis

All data recorded with publication years between 2009 and 2014 (inclusive) were analysed. The years 2009 - 2011 represent the three years before the changes in the ICN agreed at the Melbourne Congress, which came into effect on 1st January 2012; 2012–2014 represent the three years after. Although we conducted our analysis in 2017, the most recent data included in the dataset are two years old - this was to ensure that more obscure titles have had a chance to be seen by the IPNI editorial team. The lag time for some types of publications (e.g., small print-run journals and some books) can be up to a year or more. Three years after the implementation of the ICN change date gives us a valuable range of samples, because at least some of the work published in 2012 would have been already in the publication system and thus done using the previous rules; thus authors would have been unable to fully take advantage of the changes in the ICN which came into effect on 1st January of that year.

Emergence trends for authors and publications were created, in order to see if more people were participating in the publication of new vascular plant names, and if the range of places available in which to publish have expanded. To get a better view of underlying trends, a longer timescale was chosen for this part of the analysis - the full decade between 2005 and 2014 (inclusive).

We recognised that taxon-specific communities of botanists may exist, such as those working in plant families with considerable horticultural interest. To assess the degree to which these communities were using e-publication in different ways, we drilled down into the flowering plant data to collate information on the rate of take-up of e-publication in particular families. For this analysis we compared three families with considerable horticultural and collector interest – Orchidaceae (orchids), Cactaceae (cacti) and Bromeliaceae (airplants and pineapples) – with three families that are of less horticultural interest – Fabaceae (beans), Solanaceae (nightshades) and Cyperaceae (sedges). We performed the same analyses on these smaller datasets as were done for the whole dataset (see above).

### Preparation

The nomenclatural acts recorded in the IPNI database were classified into three broad groupings:

- **tax. nov.** (names of new taxa) - *tax. nov*
- **comb. nov.** (new combinations) - *comb. nov*, *stat. nov*, *comb. et stat. nov.*
- **nom. nov.** (replacement names and names at new rank) - *nom. nov.*, *nom. et stat. nov.*

SQL queries were executed against the underlying IPNI database on 2017-04-28, these were scripted in the Python programming language.

### Analyses

- **Volume of nomenclatural acts**: numbers of nomenclatural acts were grouped by publication year and citation type (to distinguish names of new taxa, new combinations, and replacement names and names at new rank.
- **Use of publication channel for all nomenclatural acts:** numbers of all nomenclatural acts were grouped by year and then by publication channel. This analysis was repeated on a per-family basis for a selected number of families, some with horticulural interest.
- **Use of any ICN changes (e-publication channel, English language diagnosis) for acts representing new names**: numbers of nomenclatural acts representing new names (*tax. nov.*) were grouped by year, and then by language and publication channel combined.
- **Authors - number active**: the unique number of authors specified as members of the publishing author team in nomenclatural acts between 2005 and 2014 were counted, broken down by year.
- **Authors - number emergent**: for all the authors active in the selected period (2005-2014), their date of emergence was calculated - this is the date when they were first recorded as a member of the publishing team of a nomenclatural act. This dataset was grouped by year of emergence to give a count for each year.
- **Publications - number active**: as per the analysis for authors described above, this is the unique number of serial publications recorded as containing nomenclatural acts published between 2005 and 2014 were counted, broken down by year. A serial publication is defined as a multi-volume work.
- **Publications - number emergent**: (as per the analysis for emergent authors described above) - for all serial publications active in the selected period, their date of emergence was calculated - this is when they were first recorded as containing a nomenclatural act. This dataset was grouped by year of emergence to give a count for each year of the study.

## Results

### Volume of nomenclatural acts

The volume of nomenclatural acts – excluding lectotypifications - has remained relatively constant (Table 1; data shown graphically in Additional file 1: Figure S1). In fact, the number of new taxa described per year (ca. 3000) has remained relatively constant since its recovery from a dip due to the Second World War (unpublished data) more than 50 years ago.

**Table 1:** Numbers of nomenclatural acts recorded in IPNI

publication_year	tax. nov.	comb. nov.	nom. nov.
2009	3022	2548	195
2010	2759	2533	166
2011	2754	3155	198
2012	2960	3611	195
2013	2806	2677	133
2014	2804	2580	384

### Use of publication channel, and description or diagnosis language

**Fig. 1:**
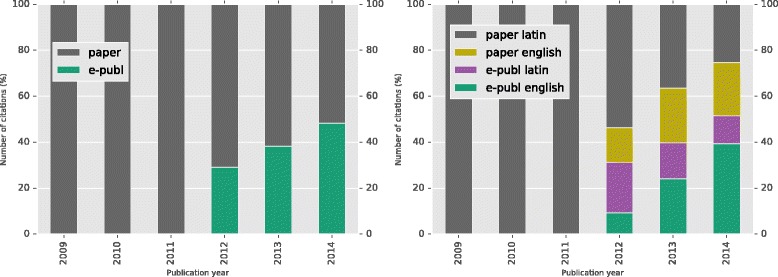
Publication channel use (all nomenclatural acts) and take-up of Code changes (plotted for tax. nov. acts only)

The use of e-publication has increased steadily from its introduction in 2012; the most recent year of the study (2014) shows that almost half (48.3%) of all acts recorded were using e-publication (Fig. 1a), data in Additional file 1: Table S1). The use of e-publication was consistent when data were analysed on a per-family basis (Additional file 1: Figure S2, data in Additional file 1: Table S1a).

When looking at only the publication of new names for taxa, the previous status quo (a description or diagnosis formed in Latin, contained within a work published on paper) is steadily diminishing, with almost three quarters (74.7%) of the new taxonomic descriptions in the final year of the study utilising at least one of the major ICN changes introduced (Fig. 1b), data in Additional file 1: Table S2). It appears that those using e-publication also more often use a diagnosis or description in English rather than Latin (Fig. 1b).

### Emergence of new authors and serial titles

The data show no sudden difference in the emergence or participation of either authors or serials after the starting date for e-publication in 2012 (indicated by the vertical line in the plot) (data in Additional file 1: Table S3). The apparent dramatic dip in the last year of the sample is likely due to the lag in discovery of nomenclatural acts published in less accessible media (e.g., small print-run local journals or books).

## Discussion

Underlying many of the hopes regarding e-publication is a recognition of a potential opportunity to overcome the so-called “taxonomic impediment” (as defined by the Convention on Biological Diversity, https://www.cbd.int/gti/problem.shtml), and to increase the communities working in taxonomy through adoption of e-dimensions to their work [28]. This means that if e-publication were a significant part of these impediments, we would expect to see more species being described, by more people, more quickly.

We structure our discussion around the principal hopes and fears regarding e-publication that were expressed during the discussions surrounding the acceptance of the changes to the ICN introduced at the XVIII IBC in Melbourne (Table 2) and which have continued to be discussed elsewhere.

**Table 2:** Hopes and fears regarding e-publication, expressed in the discussions at the XVIII IBC, Melbourne 2011

Hopes		Fears	
Rapidity	speed up publication process; biodiversity description becomes faster	Avalanche of sloppy nomenclature, leading to bad taxonomy	many new journals, little quality control
Accessibility	increase connectivity worldwide	Accessibility	lack of connectivity in the developing world; potential disenfranchisement
Inclusivity	more people in involved in description of biodiversity	Date of publication	difficulties in applying the principle of priority
Modernity	part of normal publication; improve the visibility and opinion of taxonomy	Archiving	lack of permanency; ephemeral nature of the electronic environment

Taking each of these hopes in turn:

- **Rapidity**: e-publication has not had an effect on the speed of publication of new names, and thus the rapidity of biodiversity description. The same numbers of plant species are being described every year as were before the change in the ICN (Table 1). This is likely to be the result of a number of factors, including the speed of peer-review, and the increasing numbers of specimens available for examination before decisions about the novelty of taxa can be taken. It is also abundantly evident that taxonomists now do many more things than describe and publish new taxa [29].
- **Accessibility**: e-publication as permitted in the ICN does not necessarily imply accessibility via Open Access publication. An amendment to the ICN proposed, but defeated [18] in Melbourne was to require OA publishing for all nomenclatural acts, and considerable discussion is going on in the zoological community suggesting this should be a requirement. The cost of OA publishing, however, is seen by many as restricting participation by those for the developing world, despite initiatives set up to alleviate this [1]. It is clear that accessibility needs to be associated with physical or virtual access to the work rather than any costs which may be associated with access. Accessibility is an issue for *all* types of publications, electronic and print-on-paper.
- **Inclusivity**: Our data do not show any upward trends in the numbers of authors actively publishing nomenclatural acts, nor in the number of people involved in the authorship of botanical nomenclature. Neither have we seen a decrease in either of these measures. This short term trend is only a snapshot of the longer term trend seen (for a smaller plant related dataset) by previous authors [29]. Anecdotally more authors appear to be associated with plant names, but further analysis of these trends is required. Biographical data on the authors of nomenclatural acts is not routinely collected by IPNI, and new efforts will be needed to ascertain if the community is truly changing.
- **Modernity**: This is difficult to assess with the data we have assembled. The results of our analyses show no perceptible change in numbers of publications pre and post the permitted use of e-publication, suggesting that the changes are seen as part of the normal publication process. Also discussed under modernity was the wish to improve the visibility and opinion of taxonomy - electronic publication and the use of English rather than Latin for the diagnosis or descriptions, is on the rise (see Fig. 1), and the influence of the ICN on nomenclatural practice in general can be seen in the choice of the ICZN to back-date e-publication to match the ICN starting date (1 Jan 2012) [4].

If the hopes have not been fully realised, what of the fears? The fears expressed about the acceptance of e-publication are underpinned by concerns about the potential for fragmentation of the community - either geographically or through time - and a consequent lessening in taxonomic quality. Our analysis of families with considerable horticultural interest versus those without such associated communities showed no difference in the rates of use of e-publication (see Additional file 1), so we suggest that fears regarding a fragmentation of the community based upon specialisation currently seem unrealised.

**Fig. 2:**
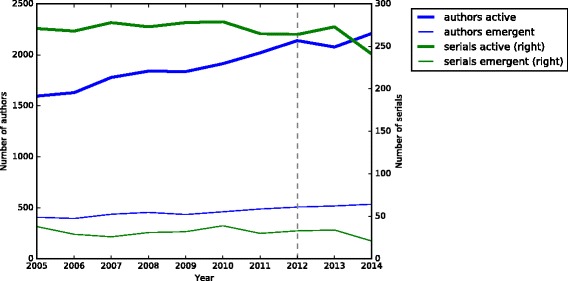
Number of authors & serials active & emergent/year (2005–2014)
- **Accessibility**: Our data cannot address the fear that e-publications will potentially be less accessible to the wider botanical community than print-on-paper publications. Anecdotally, however, it seems that the move in the publishing world from printed copies to electronic-only publication of journals has not limited access to the scientific literature. The ubiquity of internet connectivity seems only to be on the rise. We are currently assembling data to examine this aspect of publishing botanical nomenclature. Issues regarding accessibility to the literature containing nomenclatural acts will be better addressed in a separate analysis, which is more focussed on the literature itself (rather than the abstracted subset available here).
- **Date of publication**: The principle of priority is dependent upon the retrieval of an effective date of publication for any nomenclatural act, be it a new species, new combination, name at new rank or lectotypification. Over the course of the 3 years of data we assembled, there have been a handful of cases requiring investigation, these are not common and in fact are no different than any other nomenclatural problem needing investigation to resolve. It is, however, an issue that many journals still do not place the date of effective publication in the required PDF of the publication, but instead place it elsewhere, for example in the table of contents for the journal. It is imperative that botanists work with publishers to ensure that e-publications best serve future generations of botanists – this will be an on-going conversation [20] and we have really only just started.
- **Archiving**: Access to past literature is fundamental for systematics, as it is for all of science. At the current point in time - just five years after the first e-published works - we are too early to fully assess issues regarding archival storage and long term accessibility. Because archiving of works is not part of the requirements for effective publication under the ICN (for either print or e-publications), resolution of this concern does not lie in the rules of the ICN, but rather, in continued dialogue with publishers of works that contain botanical nomenclature.

- **Sloppy nomenclature**: We have not seen an avalanche of nomenclatural activity creating “bad taxonomy” since the acceptance of e-publication. The numbers of journals continuing to be active in the process of publishing botanical nomenclature has remained more or less constant (Fig. 2) and there has not been a dramatic upsurge in the establishment of new journals. Acts of “sloppy nomenclature”, such as publication that is not effective or not valid under the ICN, have also not increased since the advent of e-publication (data not shown), but longer term trends are needed.

## Conclusions

Our analysis shows that in the time frame we have analysed, three years after the implementation date for e-publication, nomenclature as applied to vascular plants continues to be in a steady state - both in terms of the number and quality of nomenclatural acts recorded, and the participation of those doing the science that results in these acts. We can therefore conclude that one of the more modest hopes - that e-publication is seen as part of the normal publication process - has been realised. In fact, we did not need to run for our lives [15]: the issues imagined have not flooded us with problems different to those perennially associated with nomenclature.

The result that the acceptance of e-publication has not elevated the rate of species description nor increased the numbers of people involved in naming new taxa means that as a community, botanists must consider other ways to speed up taxonomy. Some issues that have been raised include the large numbers of specimens now available for examination before a decision can be reached about the novelty of a taxon, the advent of a perception that molecular as well as morphological data are necessary for making a taxonomic decision, and the rigour of the peer-review process that accompanies modern publication. It still takes as long to make a decision about the identity of a specimen as it always has done, and if a botanist has 3000 specimens to look at it necessarily will take longer. Human resource issues are likely to be crucial for increasing the rate of taxonomy; our efforts perhaps should be focusing on this rather than on technological quick fixes.

It is clear that much discussion remains to be had with the publishers of nomenclature about some of the issues that have arisen, such as display of the date of effective publication, access and archiving. The results of this analysis of one part of the names governed by the *International Code of Nomenclature for algae, fungi and plants* shows that treating e-publication as an instance of publication, rather than something special to be regulated differently has been a good decision that still has the potential to help the botanical community both publish and access work describing life on Earth.

## Additional file

Additional file 1: Supplementary information contains plotted figures for data presented as tables in the text and original data tables for figures in the text. In addition, all data and plots for the per-family analysis are presented here. **Figure S1.** – Volume of nomenclatural acts by type. **Table S1.** – Data for use of publication channel (all nomenclature acts). **Figure S2.** - Use of publication channel for all nomenclatural acts - per-family breakdown. **Table S1a. **– Data for use of publication channel for all nomenclatural acts - per-family breakdown. **Table S2.** – Data for use of any Melbourne Code changes (e-publication channel, English language diagnosis) (tax. nov. acts only). **Table S3.** – Data for authors and publications – numbers active and emergent. (DOCX 76 kb)
