# Erratum to: Impact of e-publication changes in the International Code of Nomenclature for algae, fungi and plants (Melbourne Code, 2012) - did we need to “run for our lives”?

**DOI:** 10.1186/s12862-017-1009-9

**Published:** 2017-07-03

**Authors:** Nicky Nicolson, Katherine Challis, Allan Tucker, Sandra Knapp

**Affiliations:** 1 Biodiversity Informatics & Spatial Analysis, Royal Botanic Gardens, Kew, Richmond, Surrey, TW9 3AA UK; 2 IPNI, Royal Botanic Gardens, Kew, Richmond, Surrey, TW9 3AA UK; 3 Department of Computer Science, Brunel University, London, Uxbridge, UB8 3PH UK; 4 Department of Life Sciences, Natural History, Museum, Cromwell Road, London, SW7 5BD UK

## Erratum

After publication of the original article [1] the authors found that the link provided for the supporting datasets in the **‘Availability of data and materials section’**, was not working in the original article.

This was because it was redirecting to the **incorrect** link containing www: https://www.doi.org/10.6084/m9.figshare.c.3780956

The **correct** link for the supporting datasets is available here: https://doi.org/10.6084/m9.figshare.c.3780956
