# Supplementary material for: Impact of e-publication changes in the International Code of Nomenclature for algae, fungi and plants (Melbourne Code, 2012) - did we need to “run for our lives”?
Source: BMC Evol Biol. 2017 May 25;17:116. doi: 10.1186/s12862-017-0961-8 (PMC5445455; doi:10.1186/s12862-017-0961-8)
Supplement: Additional file 1: — Supplementary information contains plotted figures for data presented as tables in the text and original data tables for figures in the text. In addition, all data and plots for the per-family analysis are presented here. Figure S1. – Volume of nomenclatural acts by type. Table S1. – Data for use of publication channel (all nomenclature acts). Figure S2. - Use of publication channel for all nomenclatural acts - per-family breakdown. Table S1a. – Data for use of publication channel for all nomenclatural acts - per-family breakdown. Table S2. – Data for use of any Melbourne Code changes (e-publication channel, English language diagnosis) (tax. nov. acts only). Table S3. – Data for authors and publications – numbers active and emergent. (DOCX 76 kb) [file 12862_2017_961_MOESM1_ESM.docx]

# Additional file 1

## Volume of nomenclatural acts

### Figure SF1


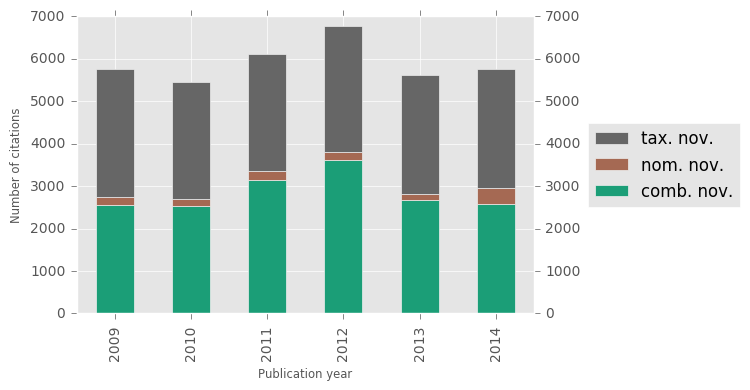


See article for table (Table 1)

Data also available as delimited data file: volume-of-nomenclatural-acts.csv

## Use of publication channel (all nomenclatural acts)

See article for plot (Figure 1(a))

### Data - table ST1

| publication_year | e-publ | paper |
| --- | --- | --- |
| 2009 | 0 | 5765 |
| 2010 | 0 | 5458 |
| 2011 | 0 | 6107 |
| 2012 | 1961 | 4805 |
| 2013 | 2142 | 3474 |
| 2014 | 2785 | 2983 |

Data also available as delimited data file: publ-channel-all-acts.csv

## Use of publication channel for all nomenclatural acts - per-family breakdown

### Figure SF2


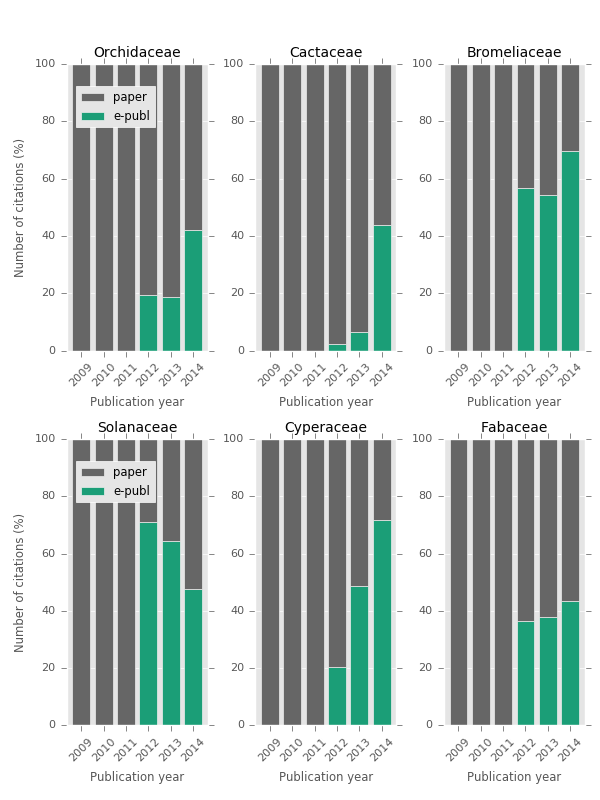


### Data - table ST1a

| family | publication_year | e-publ | paper |
| --- | --- | --- | --- |
| Bromeliaceae | 2009 | 0 | 62 |
| Bromeliaceae | 2010 | 0 | 69 |
| Bromeliaceae | 2011 | 0 | 50 |
| Bromeliaceae | 2012 | 25 | 19 |
| Bromeliaceae | 2013 | 19 | 16 |
| Bromeliaceae | 2014 | 30 | 13 |
| Cactaceae | 2009 | 0 | 69 |
| Cactaceae | 2010 | 0 | 61 |
| Cactaceae | 2011 | 0 | 180 |
| Cactaceae | 2012 | 4 | 185 |
| Cactaceae | 2013 | 9 | 129 |
| Cactaceae | 2014 | 35 | 45 |
| Cyperaceae | 2009 | 0 | 63 |
| Cyperaceae | 2010 | 0 | 96 |
| Cyperaceae | 2011 | 0 | 92 |
| Cyperaceae | 2012 | 18 | 70 |
| Cyperaceae | 2013 | 19 | 20 |
| Cyperaceae | 2014 | 97 | 38 |
| Fabaceae | 2009 | 0 | 66 |
| Fabaceae | 2010 | 0 | 34 |
| Fabaceae | 2011 | 0 | 65 |
| Fabaceae | 2012 | 8 | 14 |
| Fabaceae | 2013 | 28 | 46 |
| Fabaceae | 2014 | 13 | 17 |
| Orchidaceae | 2009 | 0 | 1430 |
| Orchidaceae | 2010 | 0 | 886 |
| Orchidaceae | 2011 | 0 | 936 |
| Orchidaceae | 2012 | 191 | 803 |
| Orchidaceae | 2013 | 170 | 736 |
| Orchidaceae | 2014 | 488 | 673 |
| Solanaceae | 2009 | 0 | 29 |
| Solanaceae | 2010 | 0 | 28 |
| Solanaceae | 2011 | 0 | 29 |
| Solanaceae | 2012 | 27 | 11 |
| Solanaceae | 2013 | 18 | 10 |
| Solanaceae | 2014 | 20 | 22 |

Data also available as delimited data file: publ-channel-all-acts-per-family.csv

## Use of any code changes (e-publication channel, English language diagnosis) (tax. nov. acts only)

See article for plot (Figure 1(b))

### Data - table ST2

|  | e-publ english | e-publ latin | paper english | paper latin |
| --- | --- | --- | --- | --- |
| 2009 | 0 | 0 | 0 | 3022 |
| 2010 | 0 | 0 | 0 | 2759 |
| 2011 | 0 | 0 | 0 | 2754 |
| 2012 | 272 | 655 | 444 | 1589 |
| 2013 | 677 | 436 | 670 | 1023 |
| 2014 | 1105 | 339 | 650 | 710 |

Data also available as delimited data file: code-changes-tax-nov.csv

## Authors & publications - numbers active & emergent

See article for plot (Figure 2)

### Data - table ST3

| year | authors active | authors emergent | serials active | serials emergent |
| --- | --- | --- | --- | --- |
| 2005 | 1596 | 409 | 271 | 38 |
| 2006 | 1630 | 396 | 268 | 29 |
| 2007 | 1779 | 438 | 278 | 26 |
| 2008 | 1841 | 456 | 273 | 31 |
| 2009 | 1836 | 435 | 278 | 32 |
| 2010 | 1914 | 462 | 279 | 39 |
| 2011 | 2020 | 489 | 265 | 30 |
| 2012 | 2140 | 508 | 264 | 33 |
| 2013 | 2077 | 519 | 273 | 34 |
| 2014 | 2210 | 536 | 241 | 21 |

Data also available as delimited data file: auth-publ-activity-emergence.csv
